# Experimental and theoretical investigation of the precise transduction mechanism in giant magnetoresistive biosensors

**DOI:** 10.1038/srep18692

**Published:** 2016-01-05

**Authors:** Jung-Rok Lee, Noriyuki Sato, Daniel J. B. Bechstein, Sebastian J. Osterfeld, Junyi Wang, Adi Wijaya Gani, Drew A. Hall, Shan X. Wang

**Affiliations:** 1 Department of Mechanical Engineering, Stanford University, Stanford, California, USA; 2 Department of Electrical Engineering, Stanford University, Stanford, California, USA; 3 MagArray Inc., 521 Cottonwood Drive, Milpitas, California, USA; 4 Department of Electrical and Computer Engineering, University of California, San Diego, USA; 5 Department of Materials Science and Engineering, Stanford University, Stanford, California, USA

## Abstract

Giant magnetoresistive (GMR) biosensors consisting of many rectangular stripes are being developed for high sensitivity medical diagnostics of diseases at early stages, but many aspects of the sensing mechanism remain to be clarified. Using e-beam patterned masks on the sensors, we showed that the magnetic nanoparticles with a diameter of 50 nm located between the stripes predominantly determine the sensor signals over those located on the sensor stripes. Based on computational analysis, it was confirmed that the particles in the trench, particularly those near the edges of the stripes, mainly affect the sensor signals due to additional field from the stripe under an applied field. We also demonstrated that the direction of the average magnetic field from the particles that contributes to the signal is indeed the same as that of the applied field, indicating that the particles in the trench are pivotal to produce sensor signal. Importantly, the same detection principle was validated with a duplex protein assay. Also, 8 different types of sensor stripes were fabricated and design parameters were explored. According to the detection principle uncovered, GMR biosensors can be further optimized to improve their sensitivity, which is highly desirable for early diagnosis of diseases.

Early diagnosis of a disease is vitally important in many situations, particularly for cancer and cardiovascular diseases. Substantial efforts have been made to identify biomarkers of diseases at their earliest stages^1^,^2^,^3^ and to improve the measurement sensitivity of such biomarkers during the past decade^4^,^5^,^6^. Magnetic biosensors, especially giant magnetoresistive (GMR) biosensors, have been shown to be amongst the most sensitive biosensors^7^,^8^. Furthermore, GMR biosensors are matrix-insensitive^7^, capable of multiplexing^9^, and temperature-insensitive^10^. Unlike autofluorescence in optical-based detection systems, GMR biosensors, also known as magneto-nanosensors, have an inherently low background signal (biological samples are naturally non-magnetic), resulting in an extremely low, femto-molar detection limit^7^. Similar to current gold standard optical techniques, GMR sensors require tagging the analyte of interest with an externally observable label, a magnetic nanoparticle in this case. These sensors have been used for many applications, including detection of DNA^11^,^12^,^13^, cancer biomarkers^7^, radiation biomarkers^14^, and cardiovascular biomarkers^15^.

While there have been substantial research efforts directed towards magnetic biosensors such as GMR^16^, magnetic tunnel junction (MTJ)^17^, and inductive sensors^18^, in conjunction with magnetic nanoparticles, there are fundamental questions concerning the optimum sensor design and particle position that remain unclear. Many studies have been conducted to understand the transduction mechanism of magnetic nanoparticles with a sensor using mathematical models and experimentally^11^,^19^. However, the GMR sensors used in previous studies were much simpler structures than current advanced GMR biosensors, particularly in terms of the sensor composition (shape and number of stripes). In addition to the evolution of sensor structure, the magnetic nanoparticles used in the previous studies were on the order of 10 nm diameters, while the later studies deploy magnetic nanoparticles on the order of 50 nm diameters^7^. Recent studies reported that magnetic nanoparticles with a diameter of 50 nm produce the largest signals under the same assay condition^13^,^15^. It has been suggested that the particles next to the stripe, not on the stripe, predominantly contribute to the signals^20^,^21^, but it had not been experimentally validated. Therefore, a new study on using advanced GMR biosensors with the magnetic nanoparticles is needed to better understand the detection mechanism of the sensors. Further understanding of the properties of magnetic particles and the transduction mechanism is imperative to further improve the sensitivity.

In this paper, we elucidated the effect of magnetic nanoparticle location on the GMR biosensors by conducting experiments and computational analysis. Different widths of sensor stripes were also investigated as one of design parameters to optimize sensor performance. The optimal design of the GMR biosensor can enhance its detection limit, which is highly desired for early diagnosis and point-of-care screening.

## Results

**Figure 1:**
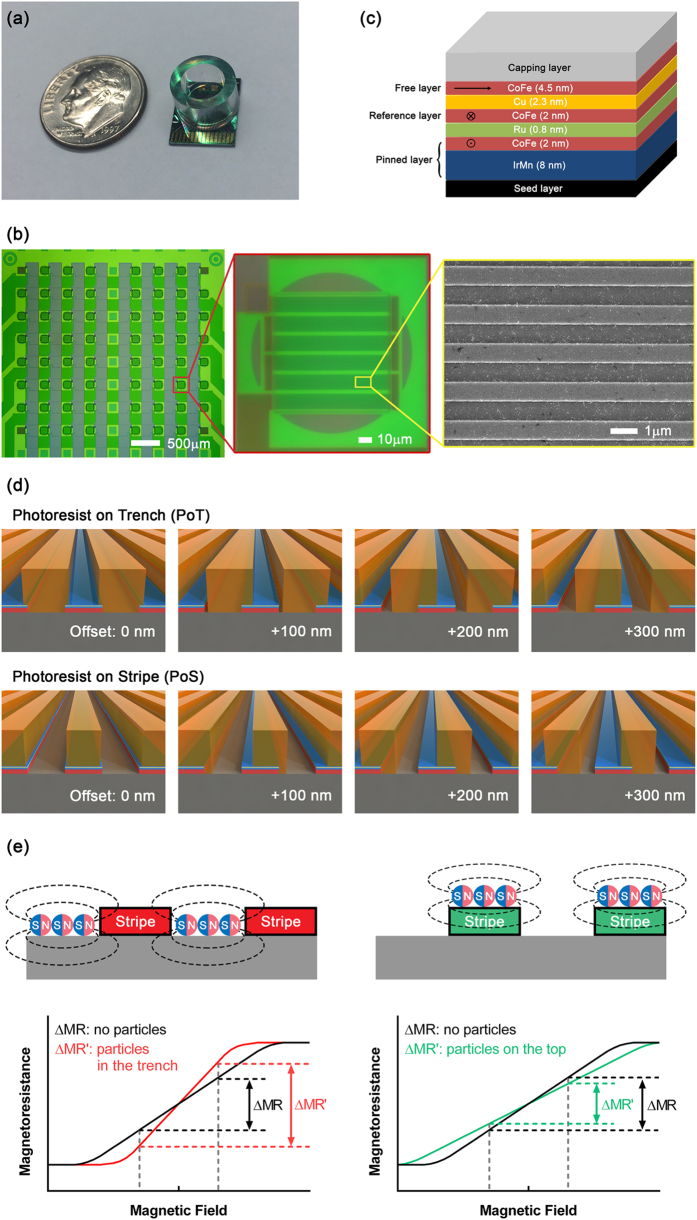
(**a**) Photograph of the GMR biosensor chip with a reaction well. Coin is US dime (17.9 mm diameter). (**b**) Left: Optical image of GMR biosensor array (8 × 8 array). Middle: Optical image of GMR biosensor element. Right: SEM image of the sensor stripes (750 nm width) with magnetic nanoparticles (50 nm diameter) bound on the surface. (**c**) Structure of the GMR biosensor stripe (not to scale). The layers of Seed layer/IrMn (8)/CoFe (2)/Ru (0.8)/CoFe (2)/Cu (2.3)/CoFe (4.5) (all thicknesses in nm) were deposited on Si/SiO_2_ substrate. The pinned layer and reference layer are coupled to maintain the magnetization in a direction. The magnetization in the free layer is aligned in the orthogonal direction due to shape anisotropy. (**d**) Different patterns of masks on GMR biosensor stripes (not to scale). The sensor stripes have a width of 750 nm (shown in blue), and the trench between two neighboring sensor stripes is 750 nm wide. The mask of Photoresist on Trench (PoT) covers the trenches with 850 nm wide photoresist patterns (shown in orange). The offset masks of +100 nm, +200 nm, and +300 nm illustrate the masks shifted from the PoT mask by 100 nm, 200 nm, and 300 nm, respectively. The Photoresist on Stripe (PoS) mask is 700 nm wide photoresist patterns located right on the top of sensor stripes. Similarly, the offset masks of +100 nm, +200 nm, and +300 nm were shifted from mask of the PoS by 100 nm, 200 nm, and 300 nm, respectively. (**e**) Schematics of magnetoresistance (MR) changes due to different locations of particles with respect to the sensor stripes. Left: The changes in MR increases with magnetic field sweeping if the particles are distributed in the trench. Right: If the particles are distributed on top of the sensor stripes, the changes in MR decrease.

An 8 × 8 array of GMR biosensor elements was fabricated with a dimension of 10 × 12 mm. A reaction well with an inner diameter of 6.35 mm was installed on the chip (Fig. 1a). The pitch between sensors is 400 μm, and each sensor element has an area of 100 × 100 μm. Each sensor consists of multiple stripes with a width of 750 nm connected in parallel and series (Fig. 1b). As shown in Fig. 1c, the GMR sensor stripes were of the spin valve type IrMn (8)/CoFe (2)/Ru (0.8)/CoFe (2)/Cu (2.3)/CoFe (4.5) (all thicknesses in nm) on seed layer^22^. Anti-ferromagnetic IrMn and CoFe layer were used as a pinned layer, and they were coupled with CoFe layer underneath the copper layer to set a reference layer. The CoFe layer on the top of copper rotates by the stray field from magnetic particles as a free layer. The relative angle between the free layer and the reference layer determines spin-dependent scattering of electrons traveling through the copper layer.

To locate the magnetic nanoparticles on specific regions of the sensor, the photoresist patterns were fabricated on the GMR sensor. A layer of negative photoresist was coated on the sensor surface using a spinner, and the spin-speed was calibrated for a photoresist thickness of 350 nm. The thickness was confirmed by atomic force microscopy (AFM) measurement (Supplementary Fig. S1). Stripe patterns with widths of 850 nm or 700 nm were exposed between the sensor stripes or on the top of the sensor stripe, respectively, in different sensor elements (Fig. 1d). The photoresist patterns in the trench between the sensor stripes (PoT) and the photoresist on the sensor stripe (PoS) patterns were shifted by 100 nm, 200 nm, and 300 nm in the transversal direction in different sensor elements, respectively. With the patterns of PoS and PoT, two opposite scenarios shown in Fig. 1e can be tested. Theoretically, these cases have the opposite behaviors in their response to an external field.

### Signal dependency on the particle location

**Figure 2:**
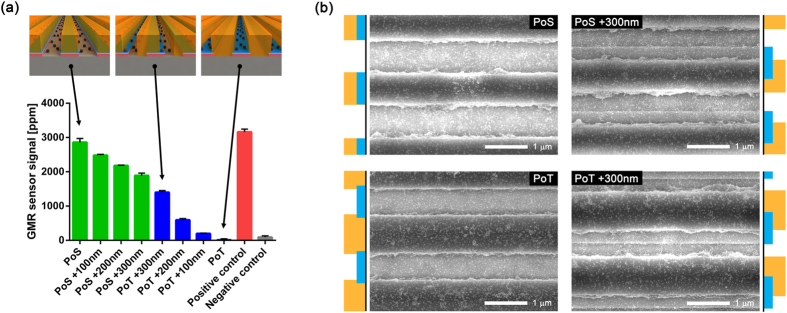
(**a**) GMR sensor signals from the sensors with different types of masks. The top three images show the relative locations of sensor stripe, photoresist mask, and the particles, approximately (not to scale) for the corresponding signals. The positive control is the sensor without any masks but coated with biotinylated BSA, while the negative control sensors are coated with BSA without any masks. The error bars are the standard deviations of identical sensors on the same chip. (**b**) SEM images of 4 different types of masks (PoS, PoS +300 nm, PoT, and PoT +300 nm) on the sensor stripes after the particles are attached. The blue bars next to the SEM images indicate the sensor stripes, and the orange bars indicate the photoresist masks.

First, the GMR sensor chip with e-beam patterned masks was measured with the reader station to study signal dependency of nanoparticle location with respect to the sensor structure. The sensor surface was coated with biotinylated bovine serum albumin (BSA), and the magnetic nanoparticles coated with streptavidin were added to the chip. Due to strong interaction between biotin and streptavidin, the magnetic nanoparticles can bind to the surface, but photoresist patterns physically separate the particles from the surface. Since the sensors with different types of masks were on the same chip, the results were monitored simultaneously and precisely compared (Fig. 2a). The sensors with PoS mask produced the highest signals, which is comparable to the positive control sensor signal. In contrast, the sensors with PoT mask generated the lowest signals, which is similar to the negative control sensor signal from sensors coated with BSA. Both positive and negative control sensors did not have any mask on them. Biotinylated BSA was immobilized on the positive control sensors as done on other sensors with masks while BSA was immobilized on the negative control sensors. As the PoS mask is shifted, the signals dropped monotonically. Analogously, the signals increased as the PoT mask is shifted. SEM images of sensors with masks were taken after the measurement was finished (Fig. 2b). The magnetic nanoparticles were properly located in between mask patterns, and their surface densities were not significantly different (Supplementary Fig. S2). All these results collectively indicate that the nanoparticles in the trench and near the sensor stripe influence the sensor signals more than particles in other locations. Even though the PoS mask was designed to have narrower patterns than the width of sensor stripe, SEM images showed that they were slightly wider than the stripe and covered the edges of the sensor stripes. This can explain why the sensors with the PoS mask produced lower signals than the positive control sensors did.

### The magnetic field from the particle at different locations

**Figure 3:**
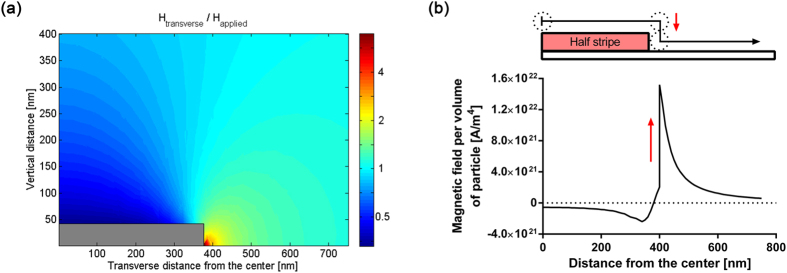
(**a**) The effective transverse magnetic field around the half of sensor stripe. The ratio of local magnetic field to the applied field was plotted in log scale. (**b**) The magnetic field from a nanoparticle at different locations on the surface normalized by the volume of the nanoparticle. The nanoparticle is moved from the center of the sensor stripe to the middle of the trench along the surface. An increase in field at 400 nm is resulted from the changes in height indicated by the red arrow. The positive sign in y axis means the same direction as an applied field.

To understand the finding, computational analysis was performed to calculate the magnetic field from a magnetic nanoparticle at different locations around the sensor stripe. In the previous studies, the magnetization of the free layer was not taken into account, and it was assumed that the thickness of the free layer is much smaller than the vertical distance of the particle from the surface^19^. To more precisely calculate the magnetic field from the nanoparticle, however, the properties of the free layer are important, especially when the nanoparticle is located in the trench. In addition, the local magnetic field due to the magnetized free layer was calculated when an external magnetic field is applied. This local magnetic field was used as the effective applied field instead of assuming an applied field is constant around the sensor stripe (Fig. 3a). Interestingly, the transverse magnetic field at the top of the sensor stripe is less than the half of the applied field, and it is at least 4 times higher near the edge of the stripe than the applied field. Since the region where the local field is the same as the externally applied field was formed around the edge of the sensor, the magnitude of the local field at the top of the sensor stripe is always less than the applied field while the local field in the trench is enhanced.

The average magnetic field from a magnetic nanoparticle over a sensor stripe was calculated using the equation written as


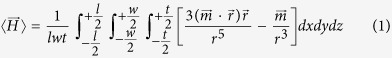


where 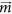 is magnetic moment of the nanoparticle, *l* is length of the sensor stripe (in x direction), *w* is width of the stripe (in y direction), and *t* is thickness of the stripe (in z direction). The calculated field was normalized by the magnetic moment of the nanoparticle (Supplementary Fig. S3). This magnetic field can be the field that determines the final signal if the particle is magnetized with the same magnitude at any location. However, the effective applied field is different at different locations. As a result, the particle is magnetized differently at different locations, which means that nanoparticles at different locations have different magnitudes of magnetization. Thus, the product of the effective applied field (Fig. 3a) and the magnetic field from a nanoparticle at the same location acting on the sensor stripe (Supplementary Fig. S3) determines the final signal due to the nanoparticle. The combination of these two factors was calculated along the center line of a nanoparticle when the nanoparticle is moving along the surface (Fig. 3b). The susceptibility of the particle, 2.08, was used in the estimation^23^, and permeability of the free layer was assumed 500 according to B-H loop measurement with bulk films. Based on the calculation, if a nanoparticle is located in the trench and right next to the edge of the sensor stripe, the magnetic field from it is 27 times higher than one located at the center of the top of the stripe. Thus, this calculation supports that the nanoparticles in the trench affect the signal more than those on the sensor stripes.

### The direction of magnetic field from the particles to the sensor stripes

**Figure 4:**
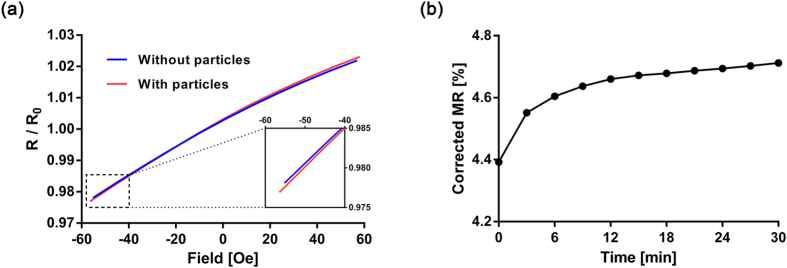
(**a**) MR curves with and without the nanoparticles measured with GMR sensors without any photoresist masks. The resistances of the sensors were obtained while an external magnetic field was swept. The blue line is the MR curve before adding nanoparticles, and the red represents the MR curve 30 mins after the nanoparticles are added. The y-axis is the resistance of the sensors divided by the nominal resistance (with no magnetic field). (**b**) Temporal changes in MR induced by bindings of streptavidin-coated nanoparticles to biotinylated BSA on the sensors. MR curves were obtained every 3 mins after adding the nanoparticles. The signal responses to the magnetic field changes were measured before adding nanoparticles, and used to correct the signals after adding the nanoparticles by the external magnetic field measured.

As described in Figs 1e and 3b, the direction of stray field from the nanoparticle should be the same as that of an applied field when the nanoparticle is located in the trench. If the signal is mainly determined by the nanoparticles located in the trench, the changes in MR upon the magnetic field sweep have to increase after the nanoparticles are attached, compared to the absence of the nanoparticles. Even though the signals obtained with double modulation scheme can tell the direction of the field from the nanoparticle^24^, another approach was used to confirm this in a more direct manner with less data processing. In the double modulation scheme, an AC magnetic field was applied externally. Even though the response of magnetization in the free layer to the applied field is much faster (usually resonance peak in GHz) than the external field applied in the experiment (210 Hz), quasi-static measurement on MR changes was conducted to avoid any effect of an alternating field. The sensors without any photoresist masks were functionalized with biotinylated BSA. The resistances of the sensors were measured while an external magnetic field was swept from −55 Oe to 55 Oe before and after adding the streptavidin-coated nanoparticles (Fig. 4a). Upon bindings of the nanoparticles via biotin-streptavidin interaction, the changes in MR became larger than those before adding the nanoparticles. This indicates that the signal is mainly affected by the nanoparticles in the trench. Even though the coil was driven by the same voltage to generate the same amount of magnetic field sweep, there were small variations in the magnitude of the external field. These variations can introduce artificial errors in changes in MR. Thus, the changes in MR were measured three times before adding the particles, and all changes in MR were corrected by the magnitude of magnetic field sweep. The MR changes were measured before adding the nanoparticles (t = 0), and every 3 minutes after adding the nanoparticles (Fig. 4b). Since MR changes increased as the nanoparticle bound to the sensors, this indicates the nanoparticles that mainly determined the signals had the same direction of magnetic field as the applied field. This again concludes that the nanoparticles located in the trench are the key particles that contribute to the signal. It also confirms that the positive sign of signals in GMR sensor signal obtained with double modulation scheme means the direction of the magnetic field from the nanoparticle is the same as the applied field.

### Detection of analytes

**Figure 5:**
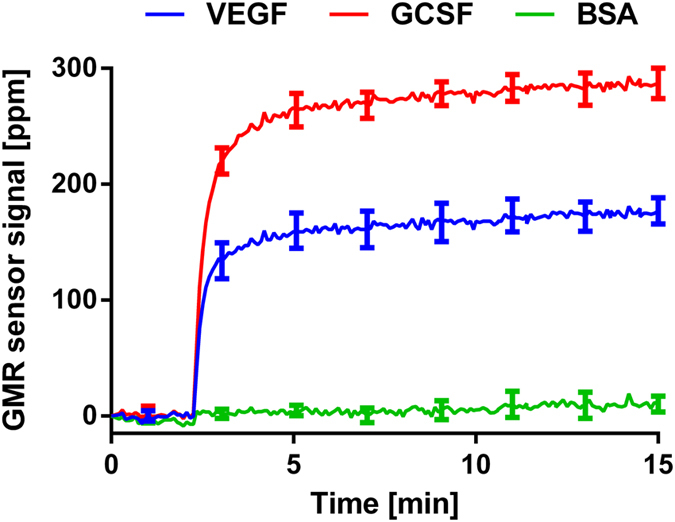
VEGF and GCSF duplex assay. Anti-VEGF and anti-GCSF antibodies were immobilized on different sensors with quadruplicates. The sample containing VEGF at 500 pg/ml and GCSF at 500 pg/ml was measured. The nanoparticles were added to the chip at 2 min. The extra distance between the nanoparticles and the surface still produced the same positive signals with respect to the baseline signal (before adding the nanoparticles). BSA signals were the same as the baseline after adding the nanoparticles. The error bars are the standard deviations of 4 identical sensor signals.

Since the sandwich assay requires a pair of antibodies surrounding the analyte of interest, it increases the height of nanoparticles up by 10 to 20 nm compared to just biotinylated BSA-coated sensors. To confirm that the effect of this extra distance above the surface does not alter the direction of the final field, a duplex protein assay was performed to detect vascular endothelial growth factor (VEGF) and granulocyte colony-stimulating factor (GCSF). The recombinant proteins of VEGF and GCSF at 500 pg/ml spiked in phosphate buffered saline (PBS) were measured with the duplex assay. In the assay, the positive sign of signals with respect to the baselines were obtained from the sensors (Fig. 5). Since we already showed that the positive sign in signals obtained with double modulation scheme means the nanoparticles located in the trench mainly determine the signals, the additional distance between the nanoparticle and the surface did not change the direction of the magnetic field detected by the sensor stripes. In addition, when the applied field was increased without adding the nanoparticles in a separate experiment, the signal increased with respect to the baseline. This also confirms that the positive sign of signal in the double modulation scheme indicates that the field applied to the sensor increases.

### Different sensor designs

**Figure 6:**
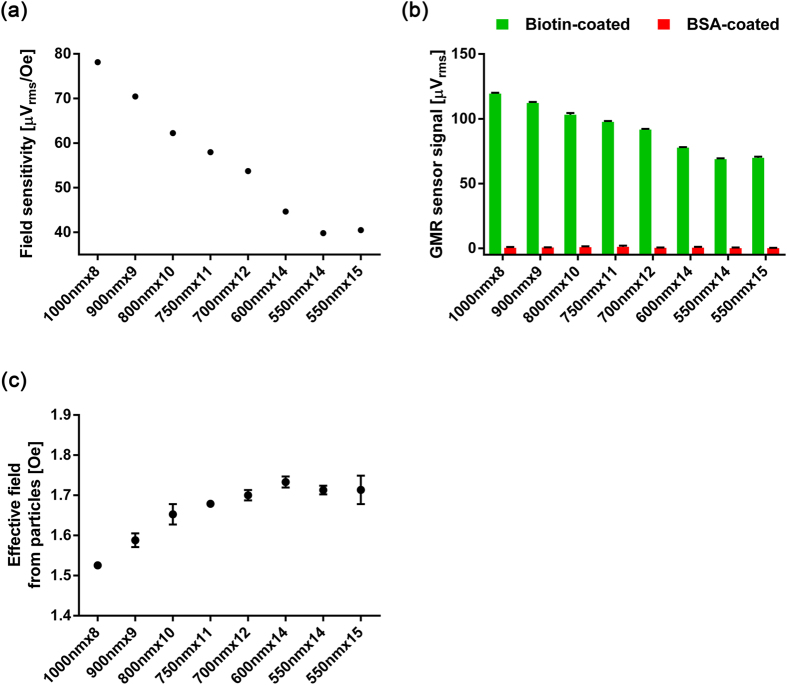
(**a**) Field sensitivity of the stripes with different widths without nanoparticles. The data points represent the average value from 4 identical sensors. (**b**) Raw signals from the sensors with different width stripes after adding the nanoparticles. The green bars are signals from biotinylated BSA-coated sensors, and the red bars are from BSA-coated sensors. (**c**) The effective field detected by different sensor types. The error bars are the standard deviations of 4 identical sensors.

To test the finding with different designs of sensor stripes and investigate the effect of stripe width, sensors with different stripe widths were fabricated to have similar footprint (100 × 100 μm) and overall sensor resistance (1.8 kOhms). Field sensitivities of 8 different types of sensor stripes were measured before adding the magnetic nanoparticles (Fig. 6a). As the sensor stripes became wider, shape anisotropy field in the free layer was expected to be reduced because the lengths of sensor stripes are all the same. As a result, the magnetization of the free layer with wider width was rotated more under the same applied field, which leads to a higher field sensitivity. After adding the nanoparticles, the signals from biotinylated BSA-coated sensors and BSA-coated sensors were obtained with the double modulation scheme for each sensor type (Fig. 6b). Due to higher field sensitivity, wider sensor stripes generated higher signals with the same coverage of the nanoparticles, but they also have higher background noise. The signals from biotinlyated BSA-coated sensors were normalized by the field sensitivity to find the effect of the number of edges of the stripes (Fig. 6c). If all nanoparticles at different locations with respect to the sensor stripe equally contribute to the signals, the normalized signals should be the same across different types of sensors. However, the sensors with fewer edges of stripes showed lower effective field from the nanoparticles. This also supports that the particles near the edge of the stripe produced more signals.

## Discussion

By both experiments and computational analysis, we confirmed that the magnetic nanoparticles located between sensor stripes contribute to the sensor signals, especially near the edges of the stripes, more than those on the top of the stripes. The direction of the average stray field from the nanoparticles that affect the sensor signal was the same as that of an applied field. The detection principle was validated with both magnetic nanoparticle detection assays as well as sandwich immnoassays. Consequently, by increasing the number of stripes within the same sensor area footprint, resulting in a high probability of capturing magnetic nanoparticles near the edges of sensor stripes, we showed that the GMR biosensor sensitivity can be enhanced. Looking ahead, there are a few more sensor design parameters that can be further investigated to understand the transduction mechanism even better. For example, the ratio of spacing between stripes to the width of the stripe, the thickness of the free layer, and the thickness of passivation layer can be explored. These studies will solidify the promise of employing GMR biosensors for high sensitivity early diagnosis of diseases.

## Methods

### GMR sensor chip fabrication

Multiple layers of GMR film were deposited on the silicon wafer after an oxide layer was grown thermally on the top of the substrate. And then the film was etched to be the stripes. Electrical connections were fabricated by depositing Ta (5)/Au (300)/Ta (5) layer (all thicknesses in nm), and then 300 nm thick oxide layer was deposited to passivate the sensors^9^. Within the sensor element area, a thin 30 nm oxide layer was deposited. The measurement probe voltage was set to 0.4 V to avoid the breakdown of the thin passivation layer. Another set of GMR sensor chips was fabricated to have different sensor stripe widths to study the effects of stripe width as well as the number of stripes. This type of GMR sensor chip includes 8 different types of sensors elements with different widths of 550 (15), 550 (14), 600 (14), 700 (12), 750 (11), 800 (10), 900 (9), and 1000 (8) (widths in nm, and the number of stripes in parentheses), and 8 identical sensor elements were fabricated on the same chip for each sensor type (total 64 sensors). The multiple layers of GMR film, electrical connections, and passivation layers were the same as the regular sensor chips described above.

### E-beam patterning of masks

The GMR biosensor chip with all 750 nm wide sensor stripes was cleaned with acetone, methanol and isopropyl alcohol prior to the electron beam lithography process. Pre-baking was then conducted in 150 °C oven for 30 min to dehydrate the chip and to improve adhesion of the photoresist to the surface. Ma-N 2403 (MicroChem Corp.) was spin-coated on the chip at 2000 rpm using a resist spinner (Headway Research Inc.). The Ma-N 2403 is a negative photoresist composed of phenolic resin and bisazide. Subsequently, the chip was baked on a hot plate at 90 °C for 1 minute to dry the photoresist. Electron beam exposure was performed on an electron beam lithography system (RAITH150 EBL, Raith Nanofabrication Ltd.) with an acceleration voltage of 10 kV and a dosage of 200 μC/cm^2^. Stripe patterns with widths of 850 nm or 700 nm were exposed between the sensor stripes or on the top of the sensor stripe, respectively, for PoT and PoS. The patterns of PoT and PoS were shifted by 100 nm, 200 nm, and 300 nm in the transversal direction in different sensor elements, respectively. Some of sensors were not exposed at all to have none of mask patterns on them, and they were used as negative or positive controls. After exposure, the chip was processed in a tetramethylammonium hydroxide (TMAH)-based developer, Ma-D 525 (MicroChem Corp.), for 30 seconds at the room temperature. The development was stopped by treatment with distilled water (Invitrogen, USA) for 3 minutes followed by a drying process in nitrogen gas flow. The remaining photoresist patterns were used as masks to block the magnetic nanoparticles from binding to the sensor surface.

### Assay with e-beam patterns

On the e-beam patterned sensors, biotinylated BSA (Sigma-Aldrich, USA) was directly deposited by using a non-contact arrayer. Either BSA or biotinylated BSA was immobilized on the non-patterned sensors on the same chip as negative or positive controls, respectively. After overnight incubation, the chip was washed with rinsing buffer (PBS pH 7.4 (Invitrogen, USA) with 0.05% polysorbate 20 (Sigma-Aldrich, USA) and 0.1% BSA), and blocked with 1% BSA for 1 hour at the room temperature. The BSA solution was washed away with the rinsing buffer, and the chip was inserted into the reader station. The same signal processing and correction schemes were applied as described in the previous studies^10^,^25^. After all calibration steps, the magnetic nanoparticles (Miltenyi Biotec, USA) were added to the chip, and binding signals were recorded until they reached to their plateaus. The steady state plateau signals were taken as GMR sensor signals. After the measurement was completed, the chip was washed with distilled water to remove the salts and unbound nanoparticles for scanning electron microscopy (SEM) images.

### Assay with analytes

For the GMR sensor chips used to detect analytes, the sensors were functionalized with the method described in the previous study^14^. Briefly, the chips were treated with 1% poly(allylamine hydrochloride, Sigma-Aldrich, USA) for 5 minutes at the room temperature after 3 minutes of oxygen plasma cleaning. The chips were washed with distilled water and baked at 120 °C for 1 hour, followed by treatment with 2% poly(ethylene-alt-maleic anhydride, Sigma-Aldrich, USA) for 5 minutes. The chips were washed again with distilled water, and activated with a mixture of Hydroxysuccinimide (NHS, Sigma-Aldrich, USA) and 1-ethyl-3-(3-dimethylaminopropyl)carbodiimide hydrochloride (EDC, Thermo Scientific, USA) in distilled water for 1 hour. Then, the chips were washed with distilled water, and ready for immobilizing the proteins on the surface. For detection of analytes, anti-VEGF (AF-493-NA, R&D systems, USA) and anti-GCSF (MAB414, R&D systems) antibodies were immobilized on different sensors with quadruplicates, and the chip was incubated overnight at 4 °C. The unbound antibodies were washed with the rinsing buffer, and the surface was blocked with 1% BSA for 1 hour at the room temperature. After washing the chip, recombinant VEGF (493-MV, R&D systems) and GCSF (414-CS, R&D systems) at 500 pg/ml, respectively, were added to the chip and incubated for 1 hour. The unbound recombinant proteins were washed with the rinsing buffer, and anti-VEGF (BAF493, R&D systems) and anti-GCSF (BAF414, R&D systems) biotinylated antibodies were added to the chip. After 1 hour incubation, the chip was washed with the rinsing buffer again, and inserted into the reader station. The signals were obtained as described above.

### Assay with different sensor designs

For the chip with different stripe designs, BSA and biotinylated BSA were immobilized on 4 sensors of the same type of sensor element, respectively, after NHS-EDC chemistry described above. Then, the chip was incubated overnight, and measured at 20 °C with the reader station that has temperature controlling feature with a Peltier element after the chip was blocked with 1% BSA. However, the signal correction schemes such as temperature correction and magnetoresistance (MR) correction were not applied in this experiment to obtain the raw data, and the data was further processed after the measurement was done. During the measurement, an AC field of 25 Oe was applied.

### Calculation on the magnetic field from the nanoparticle

Since the magnetization in the free layer of the sensor stripe is rotated by the y-component of average magnetic field from a nanoparticle, only the average magnetic field in y direction per magnetic moment of the particle, 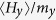, was calculated with a nanoparticle at different locations in y-z plane, using equation (1). The effective magnetic field around the sensor stripe under an external field of 50 Oe was calculated using ANSYS Maxwell software. The simulation included only the free layer of the stripe, and relative permeability of the layer used in the simulation was 500. The combination of the effective magnetic field around the sensor stripe and the averaged y-component of magnetic field from a nanoparticle at the same location produces the magnetic field experienced by the sensor stripe per unit volume of the particle if the susceptibility of the nanoparticle is taken into account.

### Magnetoresistance curve measurement

The GMR sensor chip with 750 nm wide sensor stripes was functionalized with the same NHS-EDC chemistry described above, and biotinylated BSA was immobilized on all the sensors. The chip was blocked with 1% BSA, and placed in the center of a Helmholtz coil driven by a bipolar power amplifier (BOP 20-20M, KEPCO). The resistances of the sensors were measured using a four-point probe with a digital multimeter (34401A, Agilent), and the magnetic field generated by the coil was measured with a gaussmeter (Model 420, Lake Shore). Customized LabVIEW software controlled devices and recorded the signals. The driving voltage to the amplifier for the coil operation was modulated from −1 to 1 V to measure MR curves. The changes in MR due to the magnetic field sweep were measured before adding magnetic nanoparticles, and then measured every 3 minutes after adding the particles. The changes in MR were corrected by the measured magnetic field sweep, and compared between before and after adding the particles.

## Additional Information

**How to cite this article**: Lee, J.-R. *et al.* Experimental and theoretical investigation of the precise transduction mechanism in giant magnetoresistive biosensors. *Sci. Rep.* **6**, 18692; doi: 10.1038/srep18692 (2016).

## Supplementary Material

Supplementary Information
