# Supplementary material for: Experimental and theoretical investigation of the precise transduction mechanism in giant magnetoresistive biosensors
Source: Sci Rep. 2016 Jan 5;6:18692. doi: 10.1038/srep18692 (PMC4700494; doi:10.1038/srep18692)
Supplement: Supplementary Information [file srep18692-s1.pdf]

Supplementary Information

Experimental and theoretical investigation of the precise transduction mechanism in giant magnetoresistive biosensors

Jung-Rok Lee, Noriyuki Sato, Daniel J.B. Bechstein, Sebastian J. Osterfeld, Junyi Wang, Adi W. Gani, Drew A. Hall, and Shan X. Wang[*,§]

[*] Department of Materials Science and Engineering, Stanford University, Stanford, California, USA
[§]Department of Electrical Engineering, Stanford University, Stanford, California, USA

Address: 476 Lomita Mall, Room 351 McCullough Building, Stanford, CA 94305

Email address: sxwang@stanford.edu

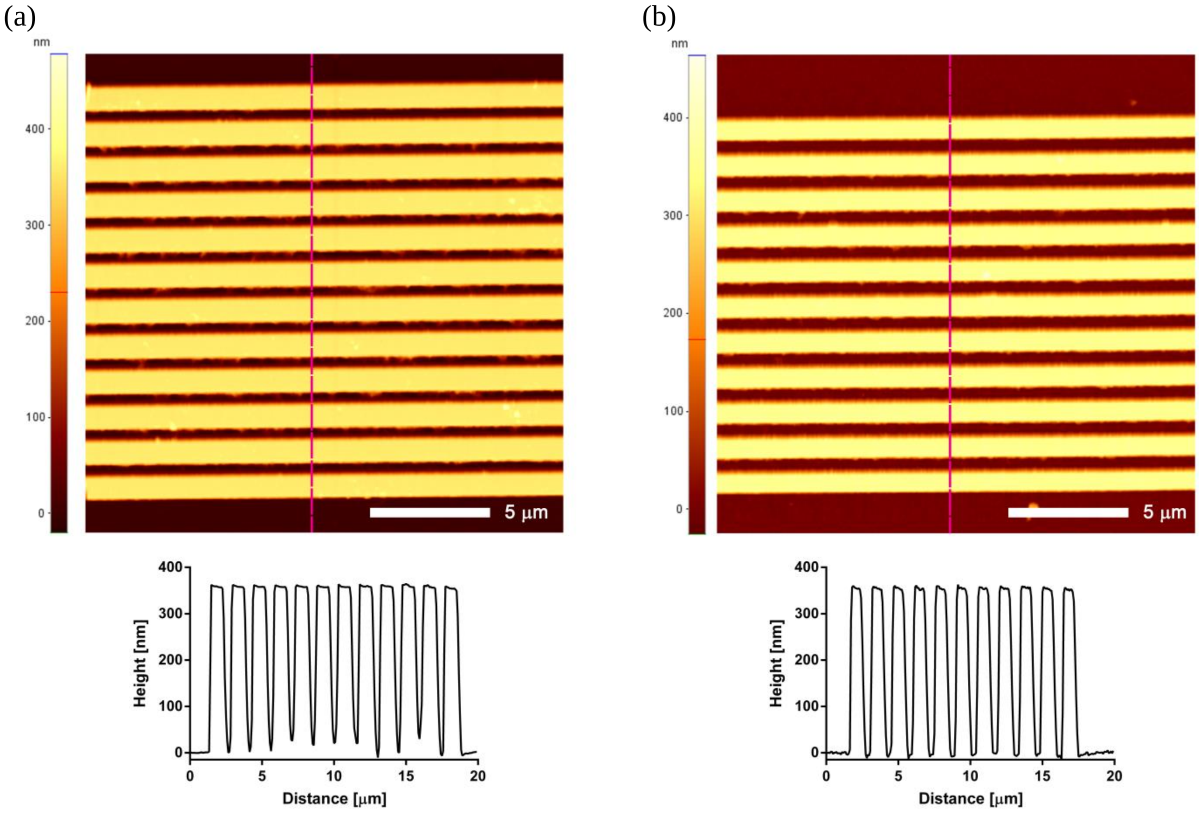

Figure S1. (a) AFM images of the PoT mask. The scan area is 20 × 20 µm, and the number of patterns is 12. The height information along the red dashed line is shown at the bottom. (b) AFM images of the PoS mask. The scan area is 20 × 20 µm, and the number of patterns is 11. The height information along the red dashed line is shown at the bottom.

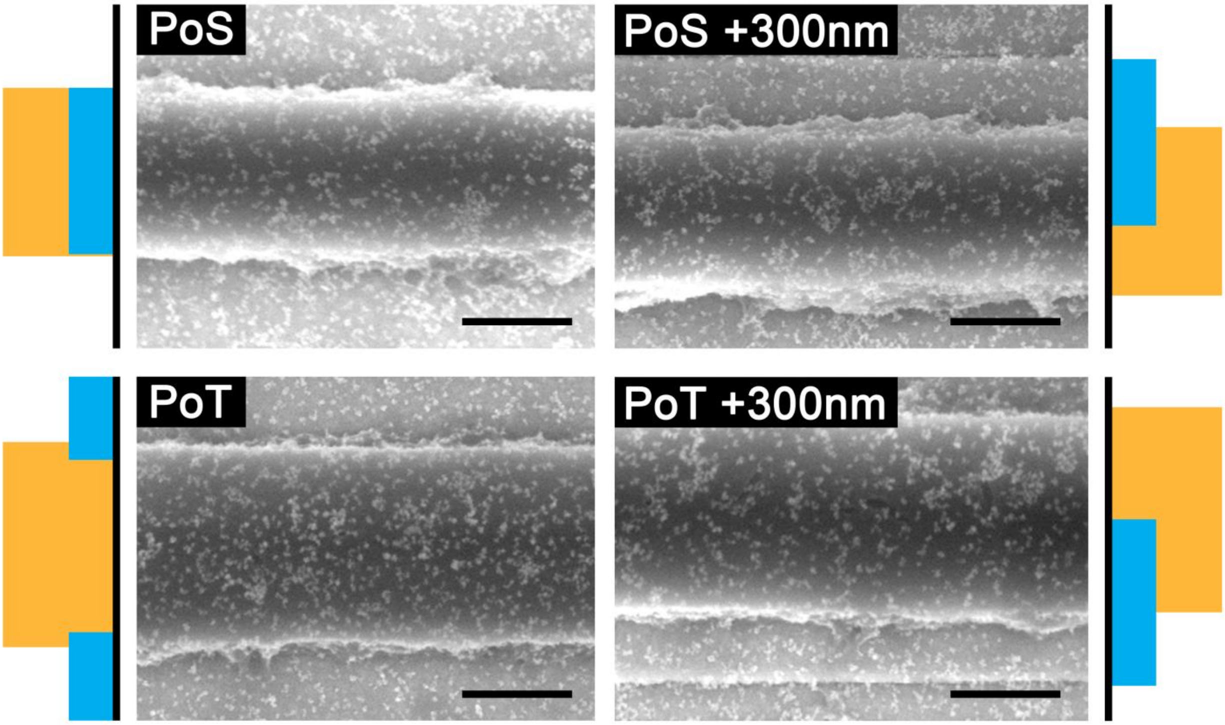

Figure S2. SEM images of 4 different types of masks (PoS, PoS +300nm, PoT, and PoT +300 nm) on the sensor stripes. The blue bars next to the SEM images indicate the sensor stripes, and the orange bars indicate the photoresist masks. Since unbound nanoparticles were washed away before SEM images were taken, only nanoparticles attached during the experiment remained on the surface. The black scale bar at the bottom right corner of each image is 500 nm.

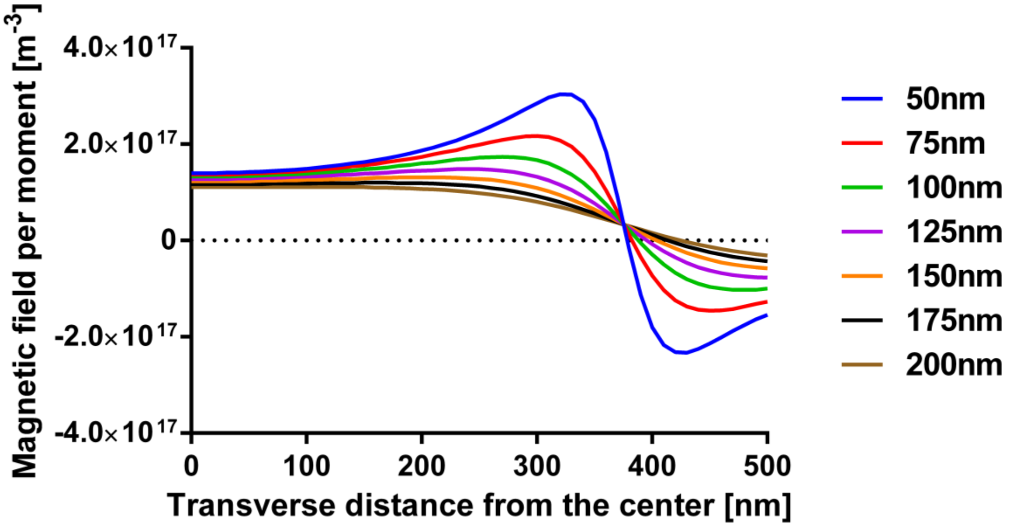

Figure S3. Magnetic field from a magnetic particle acting on the sensor stripe divided by the magnetic moment of the particle. The location of the particle is changed from the center of the sensor stripe to 500 nm in the transverse direction. The vertical position is changed from 50 nm above the sensor stripe to 200 nm with an increment of 25 nm. The positive sign in y axis means the same direction as an applied field.
